# Proteome Analysis Reveals Distinct Mitochondrial Functions Linked to Interferon Response Patterns in Activated CD4+ and CD8+ T Cells

**DOI:** 10.3389/fphar.2019.00727

**Published:** 2019-07-10

**Authors:** Marlene C. Gerner, Laura Niederstaetter, Liesa Ziegler, Andrea Bileck, Astrid Slany, Lukas Janker, Ralf L.J. Schmidt, Christopher Gerner, Giorgia Del Favero, Klaus G. Schmetterer

**Affiliations:** ^1^Department of Laboratory Medicine, Medical University of Vienna, Vienna, Austria; ^2^Department of Analytical Chemistry, Faculty of Chemistry, University of Vienna, Vienna, Austria; ^3^Joint Metabolome Facility, University of Vienna and Medical University of Vienna, Vienna, Austria; ^4^Department of Food Chemistry and Toxicology, Faculty of Chemistry, University of Vienna, Vienna, Austria

**Keywords:** metabolism, mitochondria, primary human lymphocytes, proteome profile, reactive oxygen species, T cell activation, CD4+ T cells, CD8+ T cells

## Abstract

While genetic traits and epigenetic modifications mainly encode cell type-specific effector functions, the eventual outcome is also prone to modulation by post-transcriptional regulation mechanisms. T cells are a powerful model for the investigation of such modulatory effects, as common precursor cells may differentiate either to helper CD4^+^ T cells or cytotoxic CD8^+^ cells, which elicit distinct functionalities upon TCR-stimulation. Human primary CD4^+^ and CD8^+^ T cells were purified from three individual donors and activated with anti-CD3/CD28 antibodies. Associated proteome alterations were analyzed by high-resolution mass spectrometry using a label-free shotgun approach. Metabolic activation was indicated by upregulation of enzymes related to glycolysis, NADH production, fatty acid synthesis, and uptake as well as amino acid and iron uptake. Besides various inflammatory effector molecules, the mitochondrial proteins CLUH, TFAM, and TOMM34 were found specifically induced in CD4^+^ T cells. Investigation of overrepresented conserved transcription binding sites by the oPOSSUM software suggested interferon type I inducer IRF1 to cause many of the observed proteome alterations in CD4^+^ T cells. RT qPCR demonstrated the specific induction of *IRF1* in CD4^+^ T cells only. While the interferon regulatory factor IRF4 was found induced in both T cell subtypes at protein and mRNA level, IRF9 and the type I interferon-induced proteins IFIT1, IFIT3, and MX1 were only found induced in CD4^+^ T cells. As oxidative stress enhances mitochondrial DNA-dependent type I interferon responses, the present data suggested that mitochondrial activities regulate those cell type-specific signaling pathways. Indeed, we detected mitochondrial superoxide formation predominantly in CD4^+^ T cells *via* FACS analysis with MitoSOX™ and confirmed this observation by live cell imaging with confocal microscopy. As interferon signaling regulates important features such as resistance regarding immune checkpoint blockade therapy, the present data may identify potential new targets for the efficient control of highly relevant immune cell properties.

## Introduction

T cell receptor (TCR) stimulation has a profound effect on cells resulting in the induction of multiple defense mechanisms dependent on the cell type. However, virtually all inflammatory responses somehow involve changes in protein expression, making proteome profiling a powerful tool to investigate inflammatory mechanisms as already established in our lab (Bileck et al., 2014; Slany et al., 2016; Bileck et al., 2017; Tahir et al., 2017). The relevance of metabolic control of diverse cell functions is becoming increasingly recognized, mainly with regard to tumor cells (Sullivan et al., 2016). Accordingly, also the immune system seems to be able to tune its metabolic needs and responses much more than previously anticipated (Buck et al., 2017). Optimal metabolic management is essential for immune cells in order to unfold their whole functionality on demand. Furthermore, metabolic cues are involved in the shaping of the response, e.g. the polarization and function of different T-helper cell subsets (Almeida et al., 2016) and can be used to manipulate immune responses (Sen, 2000). In the context of recent advances in immunotherapy, overcoming local T cell inhibition mediated *via* metabolic parameters seems to become an important strategy to improve the efficiency of checkpoint inhibitors (Kishton et al., 2017). Furthermore, numerous studies indicate that mitochondrial adaptations to metabolic stress may affect or even cause tumorigenesis (Seyfried et al., 2014), as also suggested by us in case of chronic lymphocytic leukemia (Mayer et al., 2018).

Although some proteome analysis studies on isolated T cells exist, (Mitchell et al., 2015; van Aalderen et al., 2017) a characterization of activation-induced proteome alterations including the comparison of CD4^+^ with CD8^+^ T cells has not been performed yet. Freshly isolated primary immune cells from healthy donors are typically quiescent and thus a more suitable choice than cultured cell lines, which normally proliferate and thus hardly represent the physiological conditions *in vivo*. Diverse immune cells of the lymphoid and myeloid lineage can be found in human peripheral blood. T cells constitute an important effector cell subset. Within the T cell compartment, CD4^+^ helper cells and CD8^+^ cytotoxic T cells represent main differentiation lineages with characteristic features (Ellmeier et al., 1999; Taniuchi, 2018). Although highly different in their effector functions upon TCR stimulation, CD4^+^ and CD8^+^ T cells display functional commonalities. The cells almost instantly respond to receptor-mediated stimulation, eventually resulting in the induction of protein synthesis for cytokine release and other, more specific, immunological functions. In general, high amounts of metabolic energy have to be invested to support these effects and gain full functionality, potentially supplementing oxidative phosphorylation (OXPHOS) with glycolysis, beta-oxidation, and glutaminolysis. This may also reflect in distinct changes in the proteome. To study this hypothesis, we isolated and purified CD4^+^ T cells and CD8^+^ T cells from peripheral blood. According to common practice we applied agonistic anti-CD3/CD28 antibodies for TCR stimulation (Trickett and Kwan, 2003). Label-free proteome profiling was applied in order to detect immune cell-specific alterations in metabolic enzyme expression accompanying stimulation focusing on mitochondrial proteins as well as associated signaling molecules. Real-time quantitative PCR was applied to independently support proteomics data of the TCR-stimulation-associated transcriptional regulation of selected molecules. As mitochondria derived superoxide anions are considered as highly relevant for stress signaling (Adler et al., 1999) as well as cancer formation (Galadari et al., 2017), we investigated the cell type-specific superoxide anion formation in T cells using FACS and live cell imaging.

## Materials and Methods

### Ethical Considerations, Cell Isolation, and Culture

The study was approved by the local Ethics Committee of the Medical University of Vienna (EC number EK 1150/2015). Peripheral blood samples were provided by the Austrian Red Cross (Vienna, Austria) upon informed written consent of the donors. Peripheral blood mononuclear cells (PBMCs) were isolated by standard Ficoll-Paque centrifugation. After isolation, PBMCs were washed in 1× PBS + 0.5% FCS + 2mM EDTA and stained with monoclonal antibodies against human CD4 (clone SK3, PerCP conjugated; BD, Franklin Lakes, NJ) and human CD8 (clone SK1, APC-Cy™7 conjugated; BD). CD4^+^ and CD8^+^ lymphocytes were FACS-sorted on a FACSAria™ Fusion (BD). Purity was assessed by flow cytometric analyses using monoclonal antibodies against human CD3 (clone OKT3, eFluor 450 conjugated; Invitrogen by Thermo Fisher Scientific, Waltham, USA) and found to be above 96.5% for CD4^+^ T cells and above 98% for CD8^+^ T cells.

### Cell Culture and Stimulation of Primary Immune Cells

All functional assays were performed in IMDM (Gibco by Thermo Fisher Scientific) supplemented with 10% fetal calf serum (Gibco), 10 µg/ml gentamycin, and 1.25 µg/ml amphotericin B (both Sigma-Aldrich, St. Louis, MO). All cells were cultured in six-well flat bottom plates. T cells were stimulated with anti-CD3/CD28 coated microbeads (Invitrogen, cells to beads ratio = 2:1).

### Sample Processing for Proteome Analysis

To obtain the cytoplasmic fraction, washed cells were lysed with mechanical shear stress and an isotonic lysis buffer (10mM HEPES/NaOH, pH 7.4, 0.25M sucrose, 10mM NaCl, 3mM MgCl_2_, and 0.5% Triton X-100) supplemented with protease inhibitors (pepstatin, leupeptin, aprotinin, each 1 µg/ml, and 1mM PMSF; all Sigma-Aldrich). Nuclei were removed via centrifugation (2,200 × *g*, 5 min, +4°C). The cytoplasmic proteins were precipitated overnight with ice-cold ethanol at −20°C. All fractionation steps were performed on ice. The precipitated proteins were digested *via* a modified filter-aided sample preparation protocol (FASP), as previously described (Mayer et al., 2018). In short, 20 µg of protein was loaded onto a 10 kD molecular weight cut-off filter (Pall, Vienna, Austria). After reduction with dithiothreitol and alkylation with iodoacetamide (all Sigma-Aldrich), the protein digestion was achieved by applying trypsin/Lys-C Mix (MS grade; Promega Corporation, Madison, WI, USA) for 16 and 4 h, respectively. The eluted peptide solution was dried *via* vacuum centrifugation and stored at −20°C until further analysis.

### LC-MS/MS Analysis

Dried peptides were reconstituted in 5 µl 30% formic acid, containing four synthetic peptides [Glu1-fribrinopeptide B, EGVNDNEEGFFSAR; M28, TTPAVLDSDGSYFLYSK; HK0, VLETKSLYVR; HK1, VLETK(ε-AC)SLYVR] for quality control. The samples were further diluted with 40 µl mobile phase A (97.9% H2O, 2% acetonitrile, 0.1% formic acid). Peptides were analyzed with a Dionex UltiMate 3000 Nano LC system coupled to a Q Exactive Orbitrap mass spectrometer, equipped with a NanoSpray Ion Source (Thermo Fisher Scientific, Austria). Preconcentration of the peptides was done on a C18 2 cm × 100 μm precolumn and LC separation was performed with a 50 cm × 75 μm PepMap100 analytical column (Thermo Fisher Scientific), at a flow rate of 300 nl/min and injection volume of 10 µl. Gradient elution of the peptides was achieved by increasing the mobile phase B (79.9% acetonitrile, 20% H2O, 0.1% formic acid) from 8% to 40%, with a total chromatographic run time of 135 min including washing and equilibration. Mass spectrometric resolution on the MS1 level was set to 70,000 (at m/z = 200) with a scan range from 400 to 1,400 m/z. The 12 most abundant peptide ions were selected for fragmentation at 30% normalized collision energy and analyzed in the Orbitrap at a resolution of 17,500 (at m/z = 200).

### Proteomics Data Analysis

Data were analyzed with small changes in settings as previously described (Mayer et al., 2018). In short, raw data were subjected to the freely available software MaxQuant (version 1.5.2.8) (Cox and Mann, 2008; Cox and Mann, 2012) utilizing the Andromeda search engine, followed by statistical evaluation with the Perseus software (version 1.6.1.0). For the Andromeda search, a minimum of two peptide identifications, at least one of them being a unique peptide, was required for valid protein identification. The false discovery rate (FDR) was set to 0.01 both on peptide and protein level. The database applied for the search was the human UniProt database (version 06/2017, reviewed entries only) with 20,386 protein entries. For statistical data evaluation of the search files obtained from MaxQuant, the Perseus software was used. Reverse sequences and potential contaminants as well as proteins identified only by site were removed. For statistical analysis, data obtained from both biological and technical replicates were used. Label-free quantification values were logarithmized to base 2 and technical replicates averaged. Proteins were filtered for valid values, keeping only proteins that were identified in at least three measurements in at least one sample group. Evaluation of regulatory events between different samples groups was achieved by two-sided t-tests using a FDR <0.05 (permutation-based FDR calculation). For selected proteins, heatmaps representing fold changes between sample groups were generated. Heatmaps were generated by a custom R (https://www.r-project.org) script plotting the label free quantification (LFQ) values obtained from Perseus. Changes marked with a plus (+) are significant (FDR < 0.05 calculated by permutation-based test). The analysis of overrepresented transcription factor binding site was performed with oPOSSUM software 3.0 (Ho Sui et al., 2005).

### Activation Control (Flow Cytometry)

After 24 h in culture, cells were washed in PBS (Gibco) + 0.5% FCS (Gibco) + 0,05% sodium azide (Sigma-Aldrich, St. Louis, MO). Cells were stained against the following anti-human mononuclear antibodies: CD25 (clone 2A3, PE-conjugated) and CD69 (clone FN50, eFluor450-conjugated; both Invitrogen) and were incubated at +4°C for 30 min and washed once more. Cells were analyzed on a BD FACSCanto II flow cytometer and analyzed using the FlowJo software (version 10, Tree Star, Ashland OR).

### MitoSOX-Assay (Flow Cytometry)

For detection of superoxide by mitochondria, the MitoSOX™ Red mitochondrial superoxide indicator, for live-cell imaging (Molecular Probes™, Invitrogen), was used according to the manufacturers’ recommendations. In this assay, MitoSOX™ Red reagent selectively targets mitochondria and is rapidly oxidized by superoxide but not by other ROS and reactive nitrogen species (RNS), yielding a red fluorescent compound. Twenty-four hours after T cell activation, 1×10^5^ cells were stained in culture medium with 5 μM MitoSOX™ Red reagent for 10 min at 37°C and were then washed three times with warm medium. The expression of MitoSOX™ Red was quantified in the PE channel via flow cytometry.

### Cellular Reactive Oxygen Species Detection (Flow Cytometry)

For ROS detection, the DCFDA Cellular ROS Detection Assay Kit (Abcam) was used. In this assay, DCFDA (2’,7’–dichlorofluorescin diacetate), a fluorogenic dye, is deacetylated by cellular esterases and later oxidized by ROS into 2’,7’-dichlorofluorescein (DCF), a highly fluorescent compound that correlates with intracellular ROS activity and can be detected in the FITC channel. Twenty-four hours after T cell activation, 1×10^5^ cells were stained in culture medium with 20 μM DCFDA for 30 min at 37°C and were then immediately transferred on ice. Without washing, ROS levels were quantified by flow cytometry in the FITC channel.

### MitoTracker (Flow Cytometry)

For quantification of mitochondria, the MitoTracker^®^ Green FM (Invitrogen by Thermo Fisher Scientific) was used. Twenty-four hours after T cell activation, 1×10^5^ cells were washed in PBS and were stained in PBS with 50 nM MitoTracker^®^ Green FM reagent for 30 min at 37°C and were then pelleted by centrifugation and resuspended in PBS. The expression of MitoTracker^®^ Green FM was quantified in the FITC channel *via* flow cytometry.

### Mitochondrial Membrane Potential (Flow Cytometry)

For the quantification of changes in mitochondrial membrane potential, the TMRE-Mitochondrial Membrane Potential Assay Kit (Abcam, Cambridge, UK) was used. TMRE (tetramethylrhodamine, ethyl ester) is a positively charged dye that accumulates in active mitochondria due to their relative negative charge. As control, the assay also uses FCCP [carbonyl cyanide 4-(trifluoromethoxy) phenylhydrazone], which eliminates mitochondrial membrane potential and TMRE staining. Twenty-four hours after T cell activation, 1×10^5^ control cells were preincubated with 20 μM FCCP for 10 min at 37°C. All cells were then incubated with 50 nM TMRE for 20 min at 37°C. Without washing, cells were analyzed *via* flow cytometry and TMRE signal was detected in the PE channel.

### Live Cell Imaging

For the live cell imaging experiments cells were incubated with CellMask™ Deep Red plasma membrane stain (gray), MitoTracker^®^ Green FM (**Figure 4E**: green, **Figure 5A**: blue; indicated as MitoTracker) and MitoSOX™ Red mitochondrial superoxide indicator (red, indicated as MitoSOX). All the dyes were used according to the specification of the supplier (1:1,000 dilution; Molecular Probes, Life Technologies, Thermo Fisher Scientific). Staining solutions were diluted in Live Cell Imaging Solution (Molecular Probes, Life Technologies) and incubated protected from light for 20 min. At the end of the incubation, cells were rinsed twice with pre-warmed Live Cell Imaging Solution and imaged with Confocal LSM Zeiss 710 equipped with ELYRA PS. 1 using a C-Apochromat 63x/1.2 W Korr M27 objective.

### RT PCR

Four hours after T-cell activation, RNA was isolated using the RNeasy^®^ Mini Kit (Qiagen, Hilden, Germany) according to the manufacturers’ recommendations. cDNA was generated by random hexamer-primed reverse transcription. Relative transcriptional levels of the indicated genes were quantified using the Luna^®^ Universal qPCR Master Mix (New England BioLabs, Ipswich, MA, USA) on a 7900HT Fast Real-Time PCR System (Applied Biosystems, Foster City, CA, USA). Transcriptional levels of GAPDH were used as reference. The following primers were used (see **Table 1**):

**Table 1:** Sequences of used RT-Primer.

Target	Sequence 5’→ 3’
CLUH	Forward: CGAGTACCTCAAGTGCCTGAC Reverse: ACTTGAGGGGCGGGATGT
GAPDH	Forward: CGAGCCACATCGCTCAGACA Reverse: GGCGCCCAATACGACCAAAT
IL-2	Forward: ATGAGACAGCAACCATTGTAGAATTT Reverse: CACTTAATTATCAAGTCAGTGTTGAGATGA
IRF-1	Forward: gtccagcccacctctgtcta Reverse: cgctgtagactcagcccaat
IRF-4	Forward: tgctttggagaggagtttcc Reverse: ttgtctggctagcagaggttc
IRF-9	Forward: aagatggagcaggcctttg Reverse: ggctctacaccagggacaga
TFAM	Forward: tggacaaacatttaaaaaggaaagct Reverse: gctgaacgaggtctttttggt
TOMM34	Forward: gcattctacagacgggctca Reverse: tgtaggaggttgctgatgtctg

For quantification ΔCT values from the respective samples were calculated (ΔCT = CT_gene_-CT_GAPDH_) and fold expression was calculated according to the formula 2^-(ΔCT_stimulated_-ΔCT_resting_).

### Statistical Analysis (Flow Cytometry Data and RT PCR)

Data were analyzed using two-sided (paired) t-test comparing resting to activated T cells using GraphPad Prism v6. Figures were also designed using GraphPad Prism.

## Results

### Proteome Profiling of TCR-Stimulated CD4^+^ and CD8^+^ T Cells

**Figure 1:**
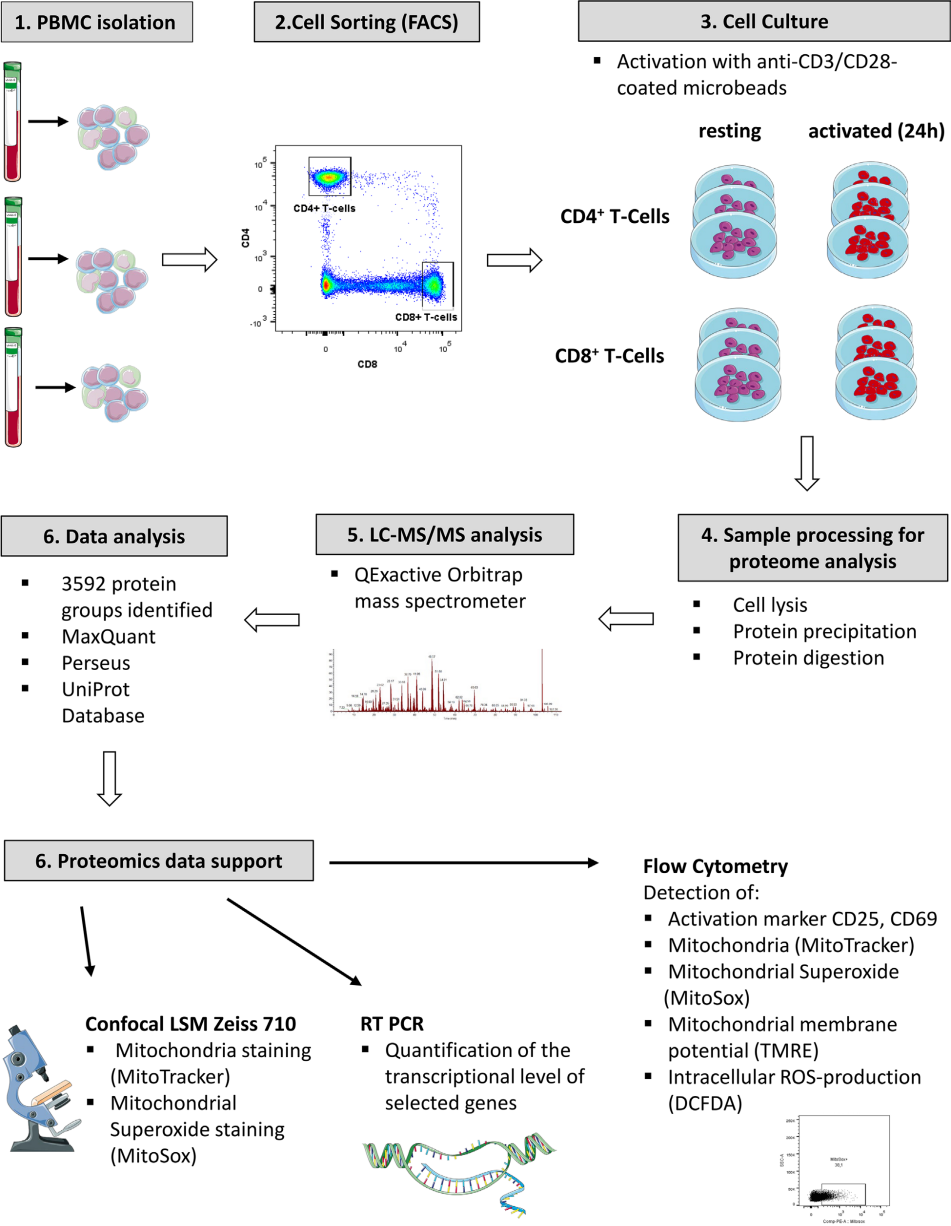
Experimental work flow.

**Figure 2:**
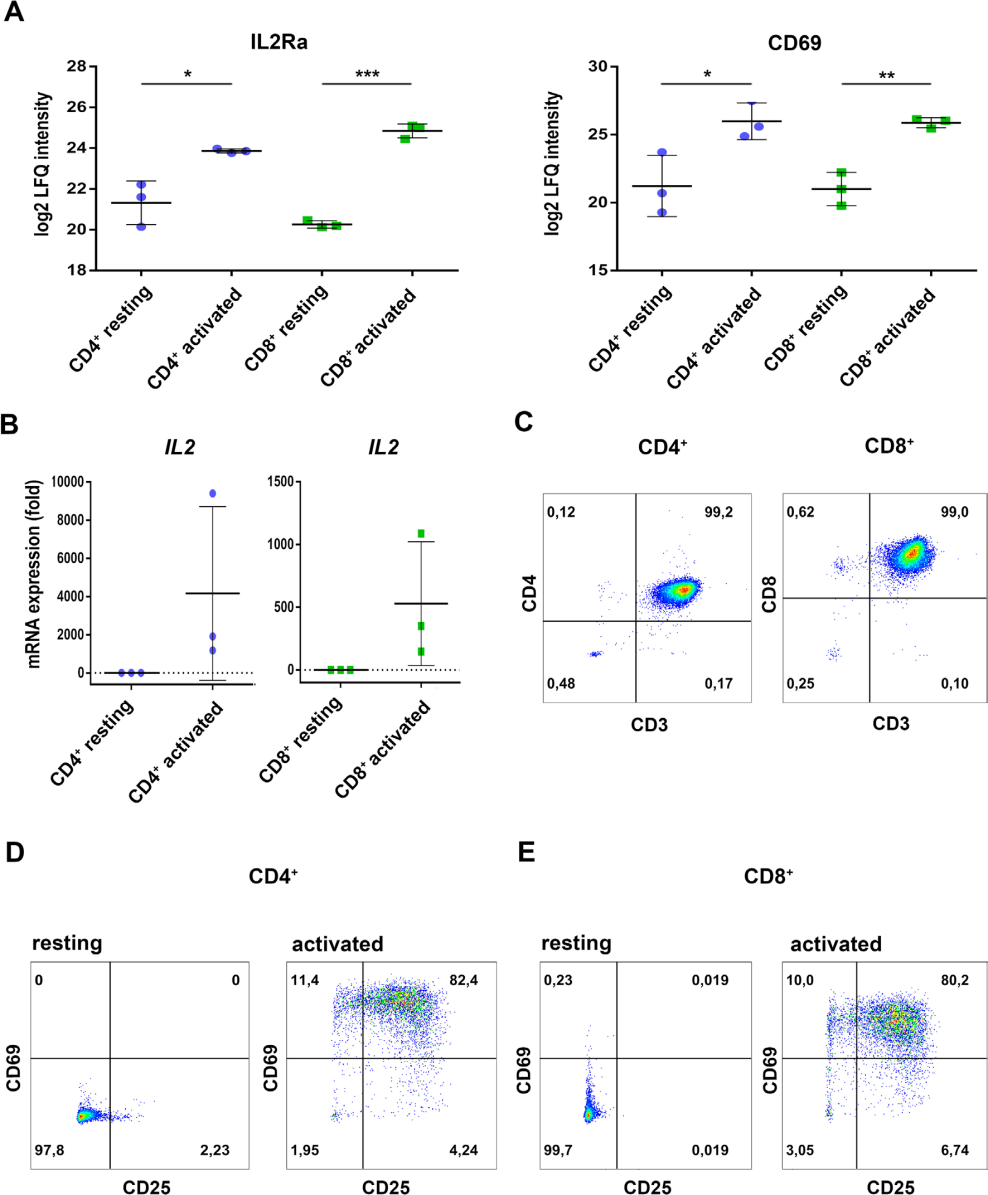
Regulation of classical activation markers on protein level, mRNA expression, and surface expression. **(A)** Purified CD4^+^ and CD8^+^ T cells were either incubated in medium (resting) or activated for 24h and proteome analyses were performed. Expression levels of the high-affinity IL2-receptor IL2a/CD25 as well as the early activation marker CD69 are shown. Data are represented as mean±SD. *P<0.05; **P<0.01; ***P<0.001 (two-sided t-test, n=3) **(B)** After 4 h of activation, IL2 mRNA-expression was analyzed by RT-PCR. Expression rates were calculated using GAPDH as a reference gene and were set relative to the expression rate in resting T cells. Data are represented as mean±SD, n=3. **(C)** Purity of the FACS-sorted CD4^+^ T cells (left) or CD8^+^ T cells (right) was analyzed by the surface expression of CD3, CD4, and CD8, data show FACS plots of one representative donor (n=3). **(D, E)** After 24 h of activation, surface expression of the early activation markers CD25 (IL2Ra) and CD69 were measured by FACS. **(D)** CD4^+^ T cells and **(E)** CD8^+^ T cells, data show FACS plots of one representative donor (n=3). Numbers in FACS plots indicate percentages of the respective populations in each quadrant.

Primary cells from three individual donors were isolated from PBMCs, purified *via* fluorescence activated cell sorting and each cell fraction was split into two aliquots (see **Figure 1** for experimental design). While one aliquot remained unstimulated under cell culture conditions for 24 h, the other aliquot was treated accordingly for TCR stimulation for the same time period. For stimulation, T cells were treated with microbeads coated with agonistic anti-CD3/CD28 antibodies. The activation markers IL2Ra (CD25) (Cerdan et al., 1992) and CD69 (Cibrian and Sanchez-Madrid, 2017) were thus found significantly induced at protein level (**Figure 2A**) and verified by flow cytometry, quantifying the percentage of activated cells as more than 90% in case of CD69 and more than 80% with regard to co-expression of both markers (**Figure 2D, E**). The gene transcript for interleukin-2 (*IL2*), another representative activation marker, was upregulated more than 100-fold in all samples (**Figure 2B**). The cell purity of primary cells was better than 96.5% for all three donors (**Figure 2C**). The cytoplasmic fraction was isolated and processed for label-free proteome profiling employing high-resolution mass spectrometry. Requiring two peptides at least per protein and applying a FDR on protein and peptide level of 0.01, we identified 3,592 protein groups; 85 and 11 proteins, respectively, were found significantly and more than two-fold up-regulated (FDR < 0.05) in CD4^+^ and CD8^+^ T cells (**Supplementary Figure S1**, **Supplementary Tables S1**, **S2**, and **S3**). In the present work we are focusing on proteome alterations related to metabolic control, while other aspects of proteome alterations including phosphoproteomics will be presented elsewhere (Janker et al., manuscript in preparation). For proteomics data support and functional analyses of mitochondria, confocal microscopy, RT PCR, and flow cytometric analyses were used (**Figure 1**).

### TCR Stimulation Induces the Expression of Metabolic Enzymes

The stimulation-induced synthesis of effector molecules such as cytokines, chemokines, adhesion molecules, and cell cycle proteins is an energy-consuming process challenging the anabolic capabilities of cells. Proteome profiling provides an unbiased approach to investigate which enzymes get up-regulated in order to support these anabolic requirements. Proteins known to critically regulate glycolysis such as hexokinase-2 (HK2) (Gershon et al., 2013) or to mediate the formation of NADH such as nicotinamide phosphoribosyltransferase (NAMPT) (Garten et al., 2015) and bifunctional methylenetetrahydrofolate dehydrogenase/cyclohydrolase (MTHFD2) (Shin et al., 2017) were found uniformly up-regulated (**Table 2**). The increased expression of the transport molecule 4F2 cell-surface antigen heavy chain (SLC3A2) points to elevated amino acid import (Ikeda et al., 2017) while the upregulated adapter molecule TNF receptor-associated factor 1 (TRAF1) indicates the general involvement of the NF kappa B signal transduction cascade (Tang et al., 2018) upon TCR stimulation (**Table 2**).

**Table 2:** Metabolic enzymes up-regulated upon TCR stimulation.

Gene name	Protein name	Accession	Fold resting CD4^+^	Q-value CD4^+^	Fold resting CD8^+^	Q-value CD8^+^
HK2	Hexokinase-2	P52789	56	6.66E-02	16	1.02E-01
NAMPT	Nicotinamide phosphoribosyltransferase	P43490	11	2.56E-02	10	8.27E-02
MTHFD2	Bifunctional methylenetetrahydrofolate dehydrogenase/cyclohydrolase	P13995	57	4.61E-02	27	1.59E-01
SLC3A2	4F2 cell-surface antigen heavy chain	P08195	61	2.90E-02	25	1.39E-01
TRAF1	TNF receptor-associated factor 1	Q13077	3	8.37E-02	13	1.27E-01
ICOS	Inducible T-cell costimulator	Q9Y6W8	10	3.49E-02	0	0
GZMB	Granzyme B	P10144	2	6.80E-01	14	2.15E-01
GBP5	Guanylate-binding protein 5	Q96PP8	72	4.63E-02	29	1.61E-01
FASN	Fatty acid synthase	P49327	24	2.56E-02	16	1.56E-01
FABP5	Fatty acid-binding protein 5	Q01469	7	2.84E-02	4	2.02E-01
HMGCS1	Hydroxymethylglutaryl-CoA synthase	Q01581	599	0.00E+00	151	9.76E-02
TFRC	Transferrin receptor protein 1	P02786	80	3.20E-02	37	6.20E-02
SLC7A5	Large neutral amino acids transporter small subunit 1	Q01650	36	4.05E-02	29	1.45E-01
DNAJA1	DnaJ homolog subfamily A member 1	P31689	16	4.58E-02	5	7.82E-01

Besides CD25 and CD69, T cells induced numerous cell type-specific molecules such as inducible T-cell costimulator (ICOS) in case of CD4^+^ cells (Simpson et al., 2010) and granzyme B (GZMB) (Pham and Ley, 1997) in case of CD8^+^ T cells (**Table 2**). Furthermore, all T cells displayed strong regulatory events related to lipid metabolism such as the induction of the fatty acid synthesis key enzyme fatty acid synthase (FASN), the fatty acid binding protein 5 (FABP5), and the key enzyme for the mevalonate pathway resulting in cholesterol synthesis hydroxymethylglutaryl-CoA synthase (HMGCS1) (**Table 2**). This was accompanied by an apparent demand for iron and amino acid import mediated *via* transferrin receptor protein 1 (TFRC) (Gammella et al., 2017) and large neutral amino acids transporter small subunit 1 (SLC7A5) (Kandasamy et al., 2018) and the induction of the protein import machinery into mitochondria *via* upregulation of DnaJ homolog subfamily A member 1 (DNAJA1) (Qiu et al., 2006) (**Table 2**).

### CD4^+^ T Cells Show a More Profound Type I Interferon Response Than CD8^+^ T Cells

**Figure 3:**
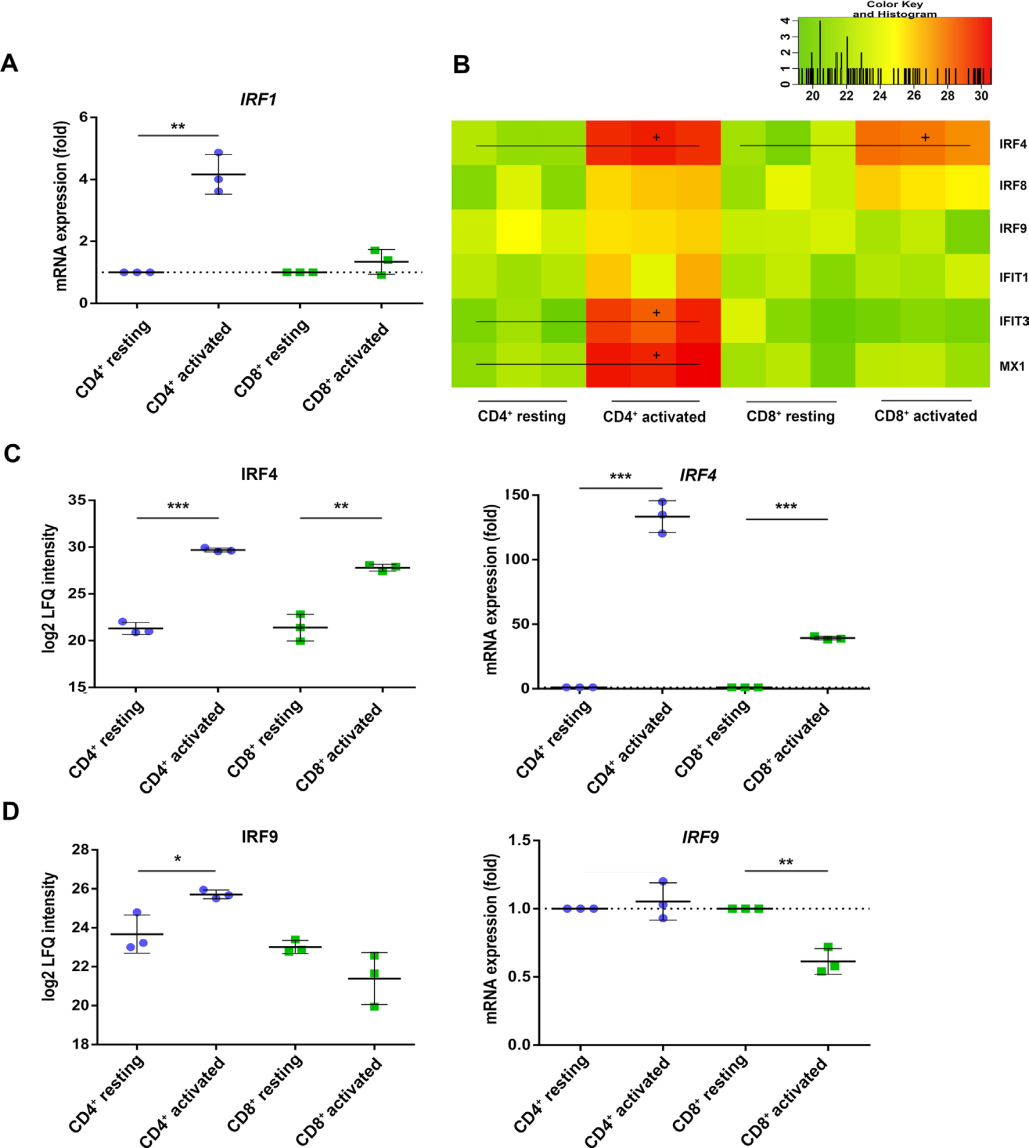
Interferon regulatory factors (IRFs) are differently regulated in activated CD4^+^ and CD8^+^ T cells. **(A)** After 4 h of activation, *IRF1* mRNA expression was analyzed by RT-PCR. Expression rate was calculated using GAPDH as a reference gene and was set relative to the expression rate in resting T cells. Data are represented as mean±SD. *P<0.05 (two-sided t-test, n=3). **(B)** Heatmap of different IRFs detected by proteome analyses of three healthy donors. Values are equal to log2 LFQ intensities. Proteins significantly up-regulated (FDR<0.05) are indicated by (+). Expression of **(C)** IRF4 and **(D)** IRF9 was detected after 24 h of activation on protein level (left) and after 4 h of activation on mRNA level (right). mRNA expression rates were calculated using GAPDH as a reference gene and were set relative to the expression rate in resting T cells. Data are represented as mean±SD. *P<0.05; **P<0.01; ***P<0.001 (two-sided t-test, n=3).

Beside the activation marker CD25 and CD69, both T cell subsets rather uniformly induced the inflammasome component guanylate-binding protein 5 (GBP5, **Table 2**) (Shenoy et al., 2012) as well as the interferon-stimulated response element (ISRE) activator interferon regulatory factor 4 (IRF4/*IRF4*) (Huber and Lohoff, 2014) at protein and mRNA level (**Figure 3C**). These observations indicate that the TCR-mediated upstream signaling occurred similarly between both T cell subsets. When investigating the proteome analysis data with the oPOSSUM software (version 3.0) (Ho Sui et al., 2005) screening for overrepresented conserved transcription binding sites, the interferon type I inducer *IRF1* together with *MYC* and *FOXF2* was suggested to cause many of the observed proteome alterations in CD4^+^ T cells, while no significant events were recorded in case of CD8^+^ T cells (**Supplementary Figure S2**). Indeed, RT PCR analysis demonstrated the specific induction of *IRF1* in CD4^+^ T cells only (**Figure 3A**). The induction of the type I interferon response proteins interferon-induced protein with tetratricopeptide repeats 1 (IFIT1), interferon-induced protein with tetratricopeptide repeats 3 (IFIT3), and interferon-induced GTP-binding protein Mx1 (MX1) in CD4^+^ T cells is shown in **Figure 3B**. IRF9 was found up-regulated in CD4^+^ T cells but rather down-regulated in CD8^+^ T cells, paralleled by *IRF9* rather unchanged in CD4^+^ T cells but significantly down-regulated in CD8^+^ T cells (**Figure 3D**). These findings demonstrate a cell type-specific interferon response upon TCR stimulation.

### Mitochondrial Functions are Specifically Altered During CD4^+^ T Cell Activation

**Figure 4:**
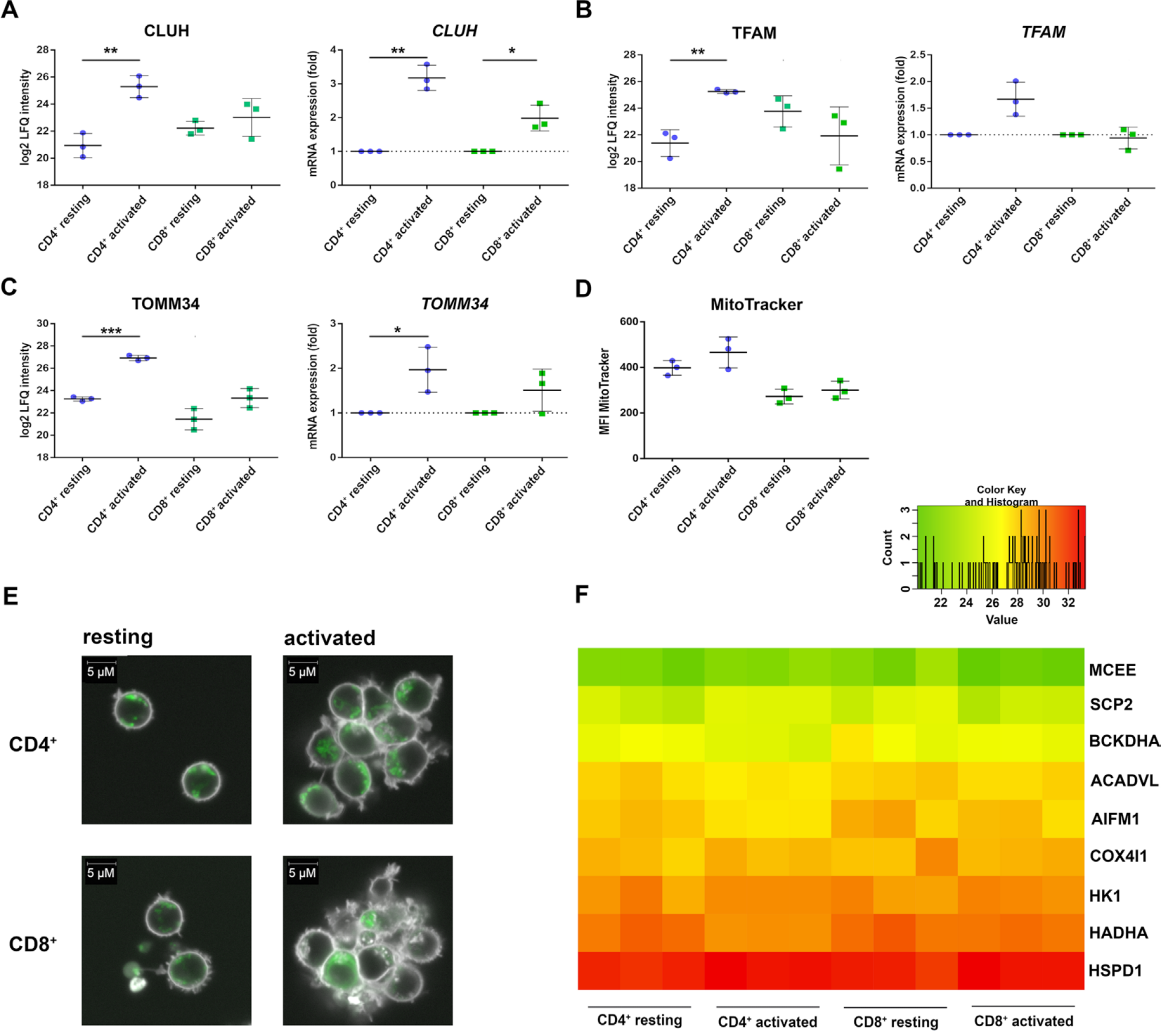
Mitochondrial proteins are differentially regulated in activated CD4^+^ and CD8^+^ T cells. **(A)** Expression of clustered mitochondrial biogenesis regulator CLUH was detected after 24 h of activation on protein level (left) and after 4 h of activation on mRNA level (right). mRNA expression rates were calculated using GAPDH as a reference gene and were set relative to the expression rate in resting T cells. Data are represented as mean±SD. *P<0.05; **P<0.01; ***P<0.001 (two-sided t-test, n=3). **(B)** Expression of mitochondrial transcription factor TFAM on protein level (left) and mRNA level (right) (n=3). **(C)** Expression of the mitochondrial transport protein TOMM34 on protein level (left) and mRNA level (right) (n=3). **(D, E)** After 24 h of activation, cells were stained with MitoTracker, to detect the amount of mitochondria in CD4^+^ and CD8^+^ T cells (n=3). **(D)** Cumulative data of MFI of MitoTracker, analyzed by FACS. Data are represented as mean±SD (n=3). **(E)** Mitochondria (green) were detected by live cell imaging; data from one donor are depicted. **(F)** Heatmap of different mitochondrial proteins detected by proteome analyses of three healthy donors. Values are equal to log2 LFQ intensities.

**Figure 5:**
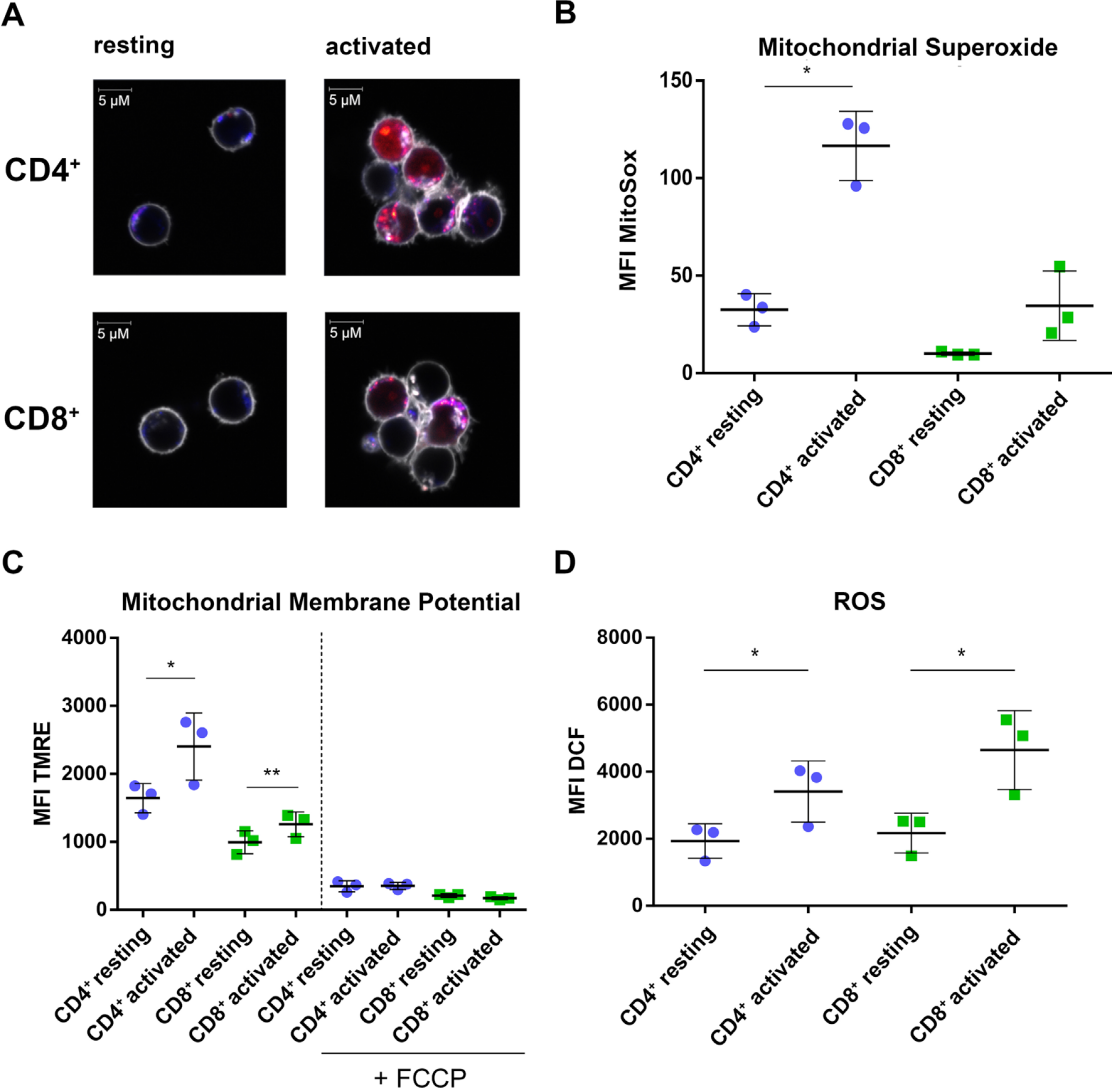
CD4^+^ T cells show increased mitochondrial activity compared to CD8^+^ T cells. **(A)** Life cell imaging of CD4^+^ and CD8^+^ T cells after staining of mitochondria (blue), mitochondrial superoxide (red), and cell membrane (gray). Data show images from one donor. **(B)** Cumulative data of MFI of mitochondrial superoxide, analyzed by FACS. Data are represented as mean±SD. *P<0.05 (two-sided t-test, n=3). **(C)** Cumulative data of MFI of TMRE as readout for mitochondrial membrane potential. Data are represented as mean±SD. *P<0.05; **P<0.01 (two-sided t-test, n=3). **(D)** Cumulative data of MFI of DCF indicating total cellular ROS production. Data are represented as mean±SD. *P<0.05 (two-sided t-test, n=3).

The CD4^+^-specific interferon pattern pointed to a specific involvement of mitochondria, which are known to contribute to an innate immune signaling *via* reactive oxygen formation accompanied by a type I interferon response (West and Shadel, 2017; Banoth and Cassel, 2018; Dhir et al., 2018). Indeed, several mitochondrial proteins including the mitochondrial biogenesis regulator clustered mitochondria protein homolog (CLUH) regulating energetic and metabolic cell status (Wakim et al., 2017), the mitochondrial transcription factor A (TFAM), and the mitochondrial import receptor subunit TOM34 (TOMM34) were found specifically induced in CD4^+^ T cells (**Figure 4A–C**). While FACS assessment of MitoTracker™ intensities altered slightly between cells and was slightly increased upon activation (**Figure 4D**), the morphology of mitochondria as well as abundance values of most mitochondrial proteins did not differ between the cells (**Figure 4E, F**). However, a predominant formation of mitochondria-derived superoxide anions in CD4^+^ T cells was observable by live cell imaging with confocal microscopy (**Figure 5A**) and proved to be statistically significant by FACS analysis of MitoSOX™-stained T cells (**Figure 5B**). This was accompanied by increased mitochondrial membrane potential values in CD4^+^ T cells (**Figure 5C**), but not by generally higher ROS levels as detected by DCF staining (**Figure 5D**). However, relevant mitochondrial ROS defense proteins such as mitochondrial superoxide dismutase (SOD2), peroxiredoxins 4, 5, and 6 (PRDX4, 5, 6), and mitochondrial thioredoxin reductase (TXNRD2) hardly differed between the cells (**Supplementary Table 3**). Abundance values of predominant lysosomal as well as peroxisomal proteins, from organelles potentially contributing to ROS (Kietzmann, 2019), did not differ significantly as well between the cells (**Supplementary Figure S3**). However, the cellular levels of cytoplasmic antioxidant proteins such as peroxiredoxins 1 and 2 (PRDX1, 2) and glutathione peroxidase-like peroxiredoxin gpx1 (GPX1) were found higher in CD4^+^ T cells compared to CD8^+^ T cells (**Supplementary Figure 3**, **Supplementary Table 3**), potentially indicating cell type specific redox management. Thus, we suggest that cell type-specific alterations of mitochondrial functions are related to the differential interferon response described above.

## Discussion

We assessed alterations in the proteome between CD3/CD28 activated CD4^+^ and CD8^+^ T cells, highlighting profound differences in metabolic regulation and the activation of interferon response factors (IRFs). Of note, several mitochondrial proteins related to maintenance and import, i.e., CLUH, TFAM, and TOMM34 were found to be up-regulated in activated CD4^+^ T-cells, while this induction was nearly absent in CD8^+^ T cells. Parallel analyses of RNA levels of the respective three genes independently confirm the specificity of the proteome analysis results and suggest that expression of these factors is regulated at the transcriptional level following T cell activation. Of note, the increase in these mitochondrial proteins was not accompanied by a substantial increase in mitochondria within the cells. The functional impact of these proteomic changes was however found at the level of mitochondrial activity. A significant increase in mitochondrial membrane potential, representing activity of the respiratory electron transport chain, as well as mitochondrial reactive oxygen species (ROS) production was observed in activated CD4^+^ T cells, while CD8^+^ T cells remained largely unaffected. Although both cell types share a similar lymphoid background and major signaling pathways following activation, obviously these signals differently impact on the cell metabolism and associated processes. While superoxide detection *via* MitoSOX™ showed marked differences between these two subsets, total ROS-levels detected *via* DCF staining were similar or showed a slightly opposite trend in CD8^+^ T cells. These observations suggest that CD8^+^ T cells may produce ROS through non-mitochondrial mechanisms, e.g., in lysosomes or peroxisomes (Kietzmann, 2019). We hypothesize that these differences in type and location of generation could be due to the differential management of ROS between the two cell types. In this regard, selective effects of superoxide and hydrogen peroxide on distinct signaling pathways have been described (Devadas et al., 2002). This notion is in line with the known inhibitory effects of antioxidants such as N-acetyl-L-cysteine on T cell responses resulting in cell death (Scheffel et al., 2016). It is thus tempting to speculate that CD4^+^ and CD8^+^ T cells use the observed selective production of either superoxide (CD4^+^) or other ROS species (CD8^+^) to shape their distinct functions, a notion that will be subject to future studies. Furthermore, these differences could be exploited to selectively target individual T cell subsets. For example, in mainly CD4^+^ T cell-driven autoimmune diseases, a selective immunosuppression of these cells would leave anti-viral and anti-tumor reactive CD8^+^ T cells intact and therefore circumvent potential side effects of global T cell suppression as currently used.

Metabolic reprogramming of immune cells upon functional activation was also recognized to be highly relevant to better understand complex disease processes such as cancer and can be regarded as model system to study these processes (Biswas, 2015). Also the present proteome analysis of TCR stimulated lymphocytes identified a large number of regulated proteins otherwise known in the context of cancer biology. The oncogenic transcription factor *MYC*, known to cause metabolic reprogramming of T cells upon stimulation (Wang et al., 2011), was indeed besides *IRF1* suggested by the oPOSSUM software to account for the presently described proteome alterations (**Supplementary Figure S2**). In addition, NAMPT (**Table 1**) is capable of inducing cancer stemness (Lucena-Cacace et al., 2017) and an invasive and drug-resistant phenotype (Ohanna et al., 2018). The important role of FASN (**Table 1**) for cancer biology (Menendez and Lupu, 2007) has triggered the development of specific inhibitors (Buckley et al., 2017). An important and specific role of mitochondria in tumor cell metabolism was already recognized decades ago (Warburg, 1956). We have observed that characteristic inflammation-related mitochondrial alterations are associated with aging and may predispose for chronic lymphocytic leukemia (Mayer et al., 2018). Here we clearly describe a cell type-specific regulation of mitochondrial activities upon acute inflammatory stimulation, which may contribute to better understand causes and consequences of such potentially critical processes. Actually, T cell activation-induced glycolysis resulting in mitochondrial reactive oxygen species generation, subsequently regulating downstream signaling cascades, was previously outlined in molecular detail (Kaminski et al., 2012). A more recent study demonstrates the relevance of CD28-mediated signaling to elicit mitochondrial fatty acid oxidation in order to achieve appropriate T cell responses (Klein Geltink et al., 2017). The generally important role of lipid metabolism for inflammation and cancer is fully recognized (Senga et al., 2018). Lipid biosynthesis has been demonstrated to coordinate mitochondrial-to-cytoplasmic stress response (Kim et al., 2016), and may thus also relate to several observations in this study. Remarkably, a link between fatty acid synthesis, cholesterol synthesis, and inflammation was described recently in macrophages (Carroll et al., 2018) but not yet in T cells. A specific difference of these mechanisms between T cell subsets has not yet been investigated up until now. Taken together, CD4^+^ T cells may present as easily accessible tool to study interventions in the above described processes. Furthermore, it remains to be assessed whether the metabolic pattern detected in CD8^+^ T cells also reflects in the situation in distinct tumors or individual patients.

Apart from metabolic differences between CD4^+^ and CD8^+^ T cells, we also found distinct IRF signatures in these genes. Interferon-induced proteins are known to mediate anti-viral effects, modulate immune functions, and regulate cell growth (Sen, 2000). A potent innate novel immune defense mechanism resulting in the release of double-stranded RNA from mitochondria was described recently to account for the induction of type I interferon response (Dhir et al., 2018). Here we describe activation-induced mitochondrial alterations accompanied by the distinct formation of superoxide anions accompanied by a marked type I interferon response characteristic for CD4^+^ T cells. This apparent link between mitochondrial function and interferon signaling may be of great relevance. Interferon signaling was found responsible for characteristic resistance properties regarding immune checkpoint blockade therapy (Benci et al., 2016). Consequently, the improved understanding of mechanisms inducing interferon responses may support patient stratification as well as anticancer therapy.

A novel combinatory antitumor therapy actually includes anti-inflammatory treatment mediated *via* dexamethasone as well as the limitation of plasma fatty acid levels *via* pioglitazone (Heudobler et al., 2018). This therapeutic approach, called anakoinosis, has been demonstrated to increase the efficiency of metronomic chemotherapy by targeting the tumor microenvironment including the immune system (Reichle and Vogt, 2008; Ugocsai et al., 2016). Here we suggest that the highly beneficial clinical effects of this therapy may be partially accounted to a potential response of T cells in addition to tumor cells to the combinatory therapy. Whether the expected alleviation of interferon signaling by the biomodulatory metronomic combination therapy is characteristic for therapy responders is currently investigated by us.

In conclusion, the present data demonstrate cell type-specific mitochondrial effector functions and downstream signaling activities characteristic for activated CD4^+^ T cells. Future studies will demonstrate whether similar mechanisms may also account for distinct properties of tumor cells and whether some of the involved molecules may represent relevant therapeutic targets.

## Author Contributions

MG, LN, LZ, AB, LJ, GF, and CG performed experiments and analyse the data. CG, KS, AS, and GF planned the research and interpreted the results. KS, RS, and CG contributed the materials. All authors contributed to the integration of the research and the preparation of the manuscript.

## Funding

This work was supported by grants from the Austrian Science Funds (FWF P29654-B30) and the Medical-Scientific Funds of the Mayor of the City of Vienna (AP18067).

## Conflict of Interest Statement

The authors declare that the research was conducted in the absence of any commercial or financial relationships that could be construed as a potential conflict of interest.

## Supplementary Material

Figure S1 Volcano plots of t-tests between **(A)** CD4+ T cells stimulated versus untreated control; **(B)** CD8+ T cells stimulated versus untreated control. **(A, B)** Permutation-based multiparameter correction was applied with an FDR of 0.05. Gray data points outside the black lines indicate significant regulations, with up-regulations on the right and down-regulations on the left side of the volcano plot.

Figure S2 oPOSSUM results. Proteomics-data of resting and activated CD4+ T cells were submitted to oPOSSUM-software allowing the detection of overrepresented conserved transcription factor binding sites in the corresponding sets of genes (depicted over the red dotted line).

Figure S3 Heatmaps of lysosomal and peroxisomal proteins detected by proteome analyses of three healthy donors. Values are equal to log2 LFQ intensities.

Table S1 Significantly upregulated proteins in activated CD4+ T cells. Data show more than two-fold up-regulated (FDR < 0.05) proteins in activated CD4+ T cells, compared to resting CD4+ T cells. Changes marked with a plus (+) are significant (FDR < 0.05).

Table S2 Significantly upregulated proteins in activated CD8+ T cells. Data show more than two-fold up-regulated (FDR < 0.05) proteins in activated CD8+ T cells, compared to resting CD8+ T cells. Changes marked with a plus (+) are significant (FDR < 0.05).

Table S3 List of identified proteins. Data show individual LFQ-values of resting and activated CD4+ and CD8+ T cells derived from three healthy donors. Differences in protein-expression are presented as “difference act vs rest” and numbers are logarithmized to base 2. “Fold resting” represents the amount of fold-regulated proteins of activated cells compared to resting cells. P-values and q-values are also depicted (see Methods).
