# Supplementary figures and images for: Proteome Analysis Reveals Distinct Mitochondrial Functions Linked to Interferon Response Patterns in Activated CD4+ and CD8+ T Cells
Source: Front Pharmacol. 2019 Jul 10;10:727. doi: 10.3389/fphar.2019.00727 (PMC6635586; doi:10.3389/fphar.2019.00727)

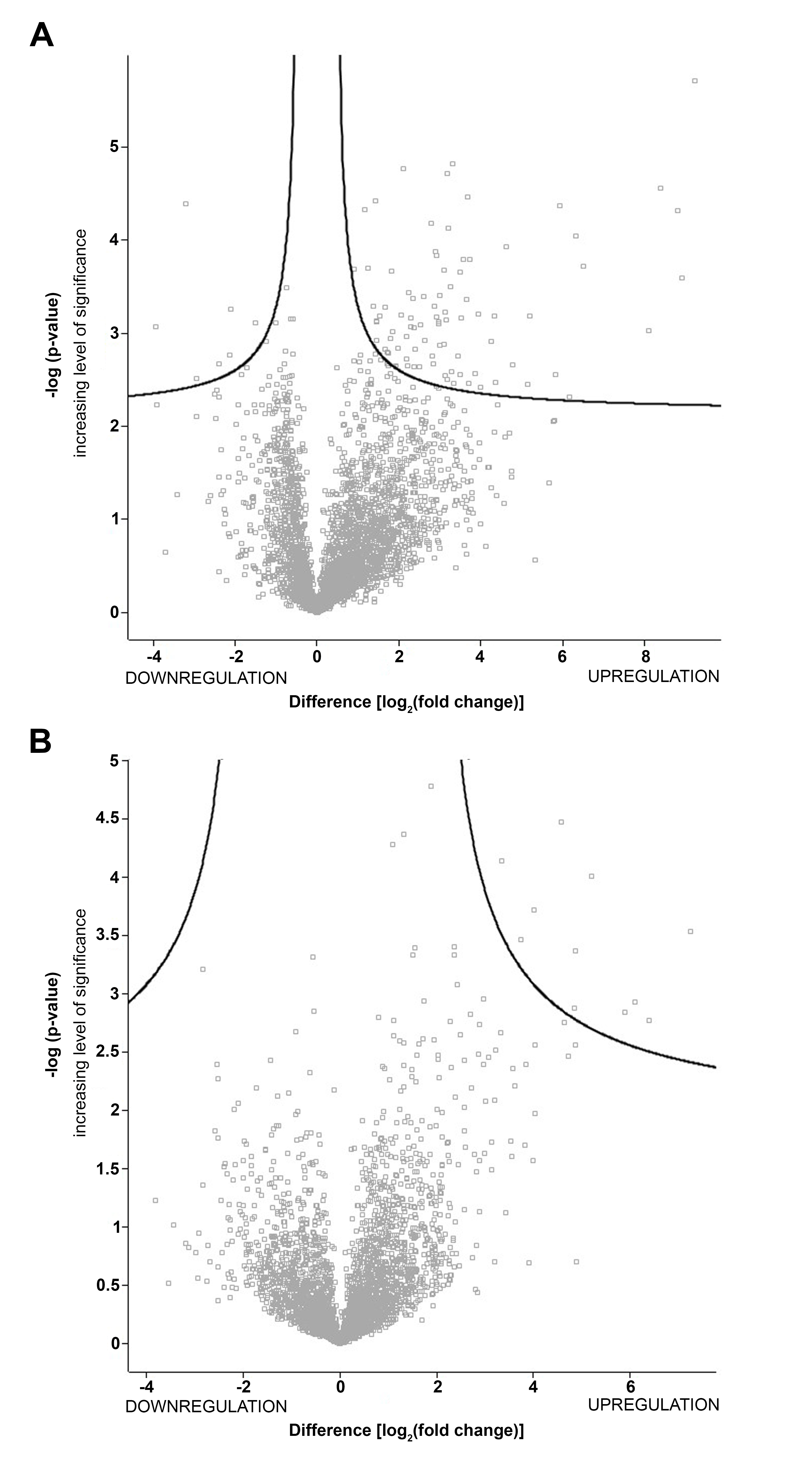

Supplement: Figure S1 — Volcano plots of t-tests between (A) CD4+ T cells stimulated versus untreated control; (B) CD8+ T cells stimulated versus untreated control. (A, B) Permutation-based multiparameter correction was applied with an FDR of 0.05. Gray data points outside the black lines indicate significant regulations, with up-regulations on the right and down-regulations on the left side of the volcano plot. [file Image_1.jpeg]

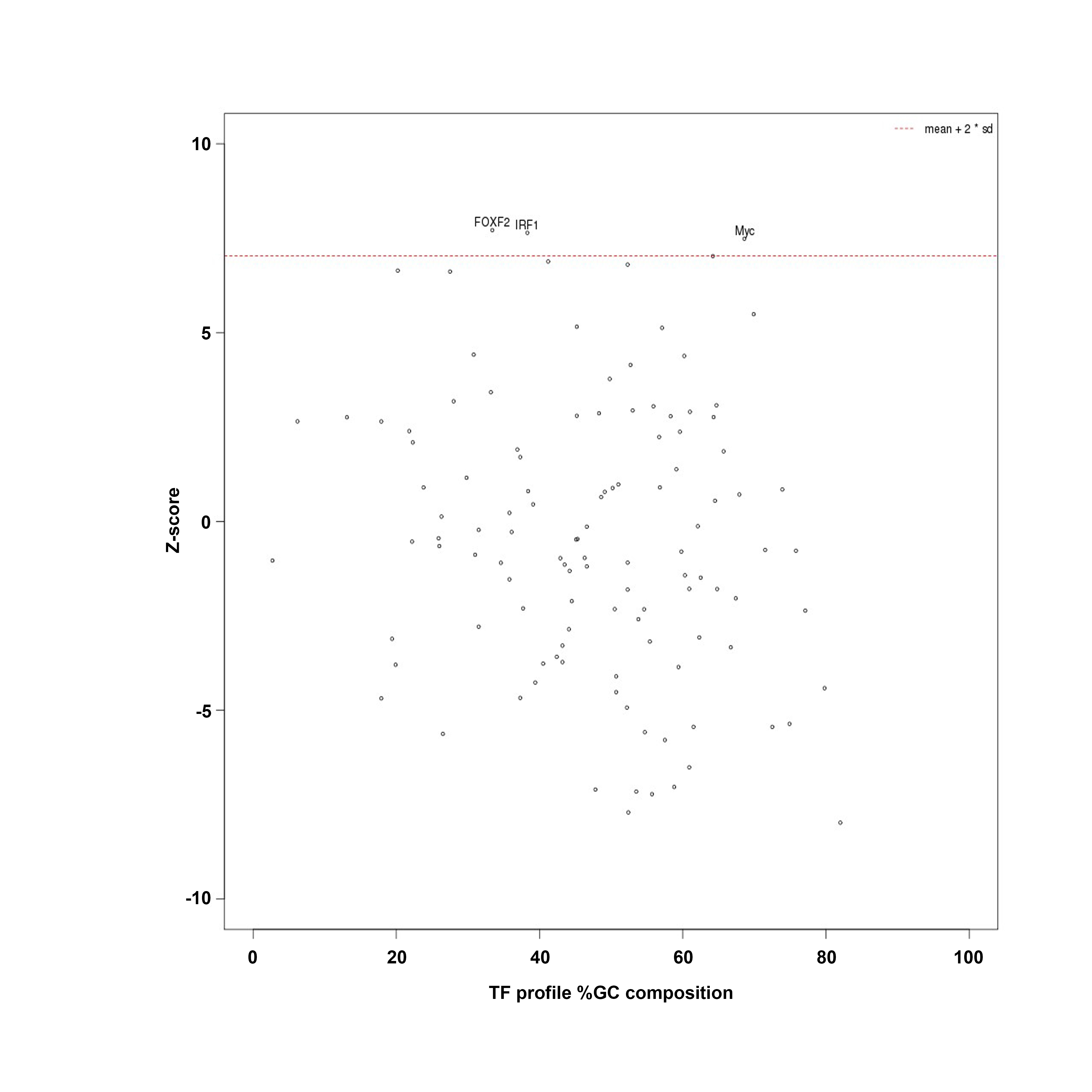

Supplement: Figure S2 — oPOSSUM results. Proteomics-data of resting and activated CD4+ T cells were submitted to oPOSSUM-software allowing the detection of overrepresented conserved transcription factor binding sites in the corresponding sets of genes (depicted over the red dotted line). [file Image_2.jpeg]

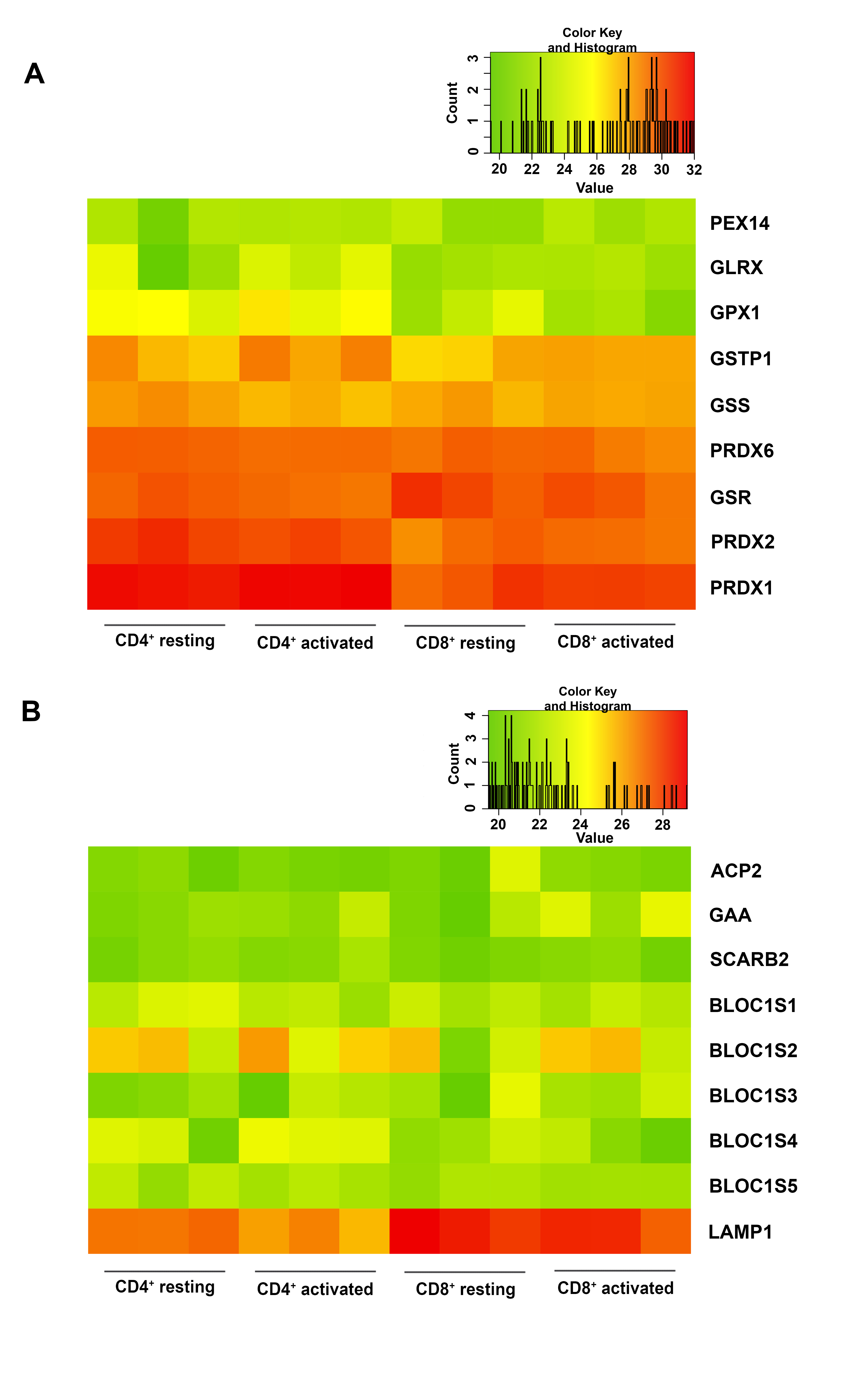

Supplement: Figure S3 — Heatmaps of lysosomal and peroxisomal proteins detected by proteome analyses of three healthy donors. Values are equal to log2 LFQ intensities. [file Image_3.jpeg]
