# Supplementary material for: Proteome Analysis Reveals Distinct Mitochondrial Functions Linked to Interferon Response Patterns in Activated CD4+ and CD8+ T Cells
Source: Front Pharmacol. 2019 Jul 10;10:727. doi: 10.3389/fphar.2019.00727 (PMC6635586; doi:10.3389/fphar.2019.00727)
Supplement: Table S1 — Significantly upregulated proteins in activated CD4+ T cells. Data show more than two-fold up-regulated (FDR < 0.05) proteins in activated CD4+ T cells, compared to resting CD4+ T cells. Changes marked with a plus (+) are significant (FDR < 0.05). [file Table_1.docx]

**Supplementary table 1: Significantly upregulated proteins in CD4^+^ T-cells**

| **Gene names** | **Protein names** | **Accession** | **Fold resting CD4^+^** | **Significant CD4 act vs rest** | **Fold resting CD8^+^** | **Significant CD8 act vs rest** |
| --- | --- | --- | --- | --- | --- | --- |
| HMGCS1 | Hydroxymethylglutaryl-CoA synthase, cytoplasmic | Q01581 | 599,17 | + | 151,41 | + |
| IFIT3 | Interferon-induced protein with tetratricopeptide repeats 3 | O14879 | 485,81 | + | 0,49 |  |
| MX1 | Interferon-induced GTP-binding protein Mx1 | P20591 | 446,65 | + | 1,87 |  |
| IRF4 | Interferon regulatory factor 4 | Q15306 | 337,07 | + | 84,36 | + |
| PSAT1 | Phosphoserine aminotransferase | Q9Y617 | 273,35 | + | 59,13 | + |
| ASNS | Asparagine synthetase [glutamine-hydrolyzing] | P08243 | 91,12 | + | 2,09 |  |
| TFRC | Transferrin receptor protein 1;Transferrin receptor protein 1, serum form | P02786 | 79,73 | + | 37,11 | + |
| GBP5 | Guanylate-binding protein 5 | Q96PP8 | 72,22 | + | 29,01 | + |
| SLC3A2 | 4F2 cell-surface antigen heavy chain | P08195 | 60,65 | + | 25,04 |  |
| MTHFD2 | Bifunctional methylenetetrahydrofolate dehydrogenase/cyclohydrolase, mitochondrial | P13995 | 57,18 | + | 26,56 |  |
| SLC7A5 | Large neutral amino acids transporter small subunit 1 | Q01650 | 36,48 | + | 29,38 | + |
| DOHH | Deoxyhypusine hydroxylase | Q9BU89 | 35,37 | + | 0,50 |  |
| TRMT10C | Mitochondrial ribonuclease P protein 1 | Q7L0Y3 | 27,28 | + | 1,93 |  |
| FASN | Fatty acid synthase;[Acyl-carrier-protein] S-acetyltransferase | P49327 | 24,55 | + | 16,40 |  |
| CLUH | Clustered mitochondria protein homolog | O75153 | 20,40 | + | 1,73 |  |
| ARPP19 | cAMP-regulated phosphoprotein 19 | P56211 | 20,18 | + | 0,38 |  |
| STAU1 | Double-stranded RNA-binding protein Staufen homolog 1 | O95793 | 19,17 | + | 0,29 |  |
| DNAJA1 | DnaJ homolog subfamily A member 1 | P31689 | 15,88 | + | 4,88 |  |
| IRF7 | Interferon regulatory factor 7 | Q92985 | 15,47 | + | 3,17 |  |
| TFAM | Transcription factor A, mitochondrial | Q00059 | 14,61 | + | 0,28 |  |
| HAT1 | Histone acetyltransferase type B catalytic subunit | O14929 | 13,19 | + | 1,00 |  |
| SSSCA1 | Sjoegren syndrome/scleroderma autoantigen 1 | O60232 | 13,05 | + | 0,97 |  |
| TOMM34 | Mitochondrial import receptor subunit TOM34 | Q15785 | 12,74 | + | 3,69 |  |
| MRPS31 | 28S ribosomal protein S31, mitochondrial | Q92665 | 12,48 | + | 0,18 |  |
| SEC23B | Protein transport protein Sec23B | Q15437 | 11,94 | + | 1,09 |  |
| ICAM1 | Intercellular adhesion molecule 1 | P05362 | 11,51 | + | 9,22 |  |
| NAMPT | Nicotinamide phosphoribosyltransferase | P43490 | 11,38 | + | 10,23 | + |
| WDR12 | Ribosome biogenesis protein WDR12 | Q9GZL7 | 11,22 | + | 9,27 |  |
| STAT3 | Signal transducer and activator of transcription 3 | P40763 | 10,92 | + | 5,10 |  |
| LARP1 | La-related protein 1 | Q6PKG0 | 10,08 | + | 1,30 |  |
| PUS7 | Pseudouridylate synthase 7 homolog | Q96PZ0 | 9,91 | + | 1,57 |  |
| EFTUD2 | 116 kDa U5 small nuclear ribonucleoprotein component | Q15029 | 9,69 | + | 4,27 |  |
| ICOS | Inducible T-cell costimulator | Q9Y6W8 | 9,53 | + | 1,00 |  |
| TRMT1 | tRNA (guanine(26)-N(2))-dimethyltransferase | Q9NXH9 | 9,24 | + | 7,96 |  |
| METTL2B | Methyltransferase-like protein 2B;Methyltransferase-like protein 2A | Q6P1Q9 | 9,09 | + | 4,78 |  |
| RPTOR | Regulatory-associated protein of mTOR | Q8N122 | 9,06 | + | 0,77 |  |
| MCM7 | DNA replication licensing factor MCM7 | P33993 | 8,96 | + | 5,93 |  |
| WARS | Tryptophan--tRNA ligase, cytoplasmic;T1-TrpRS;T2-TrpRS | P23381 | 8,84 | + | 11,55 |  |
| CTNNA1 | Catenin alpha-1 | P35221 | 8,82 | + | 1,58 |  |
| ATG4B | Cysteine protease ATG4B | Q9Y4P1 | 8,68 | + | 1,00 |  |
| TBX21 | T-box transcription factor TBX21 | Q9UL17 | 8,67 | + | 0,55 |  |
| ANKLE2 | Ankyrin repeat and LEM domain-containing protein 2 | Q86XL3 | 8,07 | + | 4,69 |  |
| DUSP4 | Dual specificity protein phosphatase 4 | Q13115 | 7,96 | + | 0,86 |  |
| KPNA2 | Importin subunit alpha-1 | P52292 | 7,91 | + | 4,23 |  |
| LGALS9 | Galectin-9;Galectin-9C;Galectin-9B | O00182 | 7,90 | + | 0,50 |  |
| NME1 | Nucleoside diphosphate kinase A | P15531 | 7,62 | + | 2,94 |  |
| HLA-DRB1 | HLA class II histocompatibility antigen, DRB1-13 beta chain | Q5Y7A7 | 7,57 | + | 0,72 |  |
| FABP5 | Fatty acid-binding protein, epidermal | Q01469 | 7,44 | + | 4,09 |  |
| PLSCR1 | Phospholipid scramblase 1 | O15162 | 7,34 | + | 1,00 |  |
| NOTCH1 | Neurogenic locus notch homolog protein 1;Notch 1 extracellular truncation | P46531 | 7,20 | + | 0,95 |  |
| HCCS | Cytochrome c-type heme lyase | P53701 | 7,06 | + | 2,07 |  |
| REL | Proto-oncogene c-Rel | Q04864 | 6,87 | + | 3,72 |  |
| E2F4 | Transcription factor E2F4 | Q16254 | 6,23 | + | 1,28 |  |
| ACSL4 | Long-chain-fatty-acid--CoA ligase 4 | O60488 | 6,07 | + | 1,80 |  |
| DYNC1H1 | Cytoplasmic dynein 1 heavy chain 1 | Q14204 | 5,81 | + | 1,53 |  |
| TRAFD1 | TRAF-type zinc finger domain-containing protein 1 | O14545 | 5,68 | + | 2,39 |  |
| RRM1 | Ribonucleoside-diphosphate reductase large subunit | P23921 | 5,23 | + | 3,24 |  |
| YBX1 | Nuclease-sensitive element-binding protein 1 | P67809 | 5,17 | + | 3,83 |  |
| HEXIM1 | Protein HEXIM1 | O94992 | 5,13 | + | 0,68 |  |
| PSMB7 | Proteasome subunit beta type-7 | Q99436 | 5,07 | + | 7,87 |  |
| ZNF24 | Zinc finger protein 24 | P17028 | 4,90 | + | 1,62 |  |
| UBE2S | Ubiquitin-conjugating enzyme E2 S | Q16763 | 4,88 | + | 1,00 |  |
| NAA16 | N-alpha-acetyltransferase 16, NatA auxiliary subunit | Q6N069 | 4,70 | + | 0,31 |  |
| POLR2A | DNA-directed RNA polymerase II subunit RPB1 | P24928 | 4,64 | + | 1,12 |  |
| BYSL | Bystin | Q13895 | 4,57 | + | 1,65 |  |
| DCTPP1 | dCTP pyrophosphatase 1 | Q9H773 | 4,55 | + | 4,13 |  |
| MAT2A | S-adenosylmethionine synthase isoform type-2 | P31153 | 4,29 | + | 4,02 |  |
| HSPH1 | Heat shock protein 105 kDa | Q92598 | 4,20 | + | 5,16 |  |
| RPS13 | 40S ribosomal protein S13 | P62277 | 3,93 | + | 2,35 |  |
| RPL18A | 60S ribosomal protein L18a | Q02543 | 3,81 | + | 1,83 |  |
| CLPX | ATP-dependent Clp protease ATP-binding subunit clpX-like, mitochondrial | O76031 | 3,60 | + | 2,88 |  |
| ACSL5 | Long-chain-fatty-acid--CoA ligase 5 | Q9ULC5 | 3,51 | + | 1,84 |  |
| MIER1 | Mesoderm induction early response protein 1 | Q8N108 | 3,44 | + | 1,65 |  |
| SERPINA1 | Alpha-1-antitrypsin;Short peptide from AAT | P01009 | 3,36 | + | 0,52 |  |
| UBAP2L | Ubiquitin-associated protein 2-like | Q14157 | 3,27 | + | 4,50 |  |
| CD74 | HLA class II histocompatibility antigen gamma chain | P04233 | 3,17 | + | 0,77 |  |
| TMX3 | Protein disulfide-isomerase TMX3 | Q96JJ7 | 3,15 | + | 0,58 |  |
| NUCB1 | Nucleobindin-1 | Q02818 | 2,90 | + | 2,84 |  |
| SHMT2 | Serine hydroxymethyltransferase, mitochondrial | P34897 | 2,75 | + | 3,67 |  |
| PDCD2L | Programmed cell death protein 2-like | Q9BRP1 | 2,74 | + | 2,52 |  |
| EIF4A1 | Eukaryotic initiation factor 4A-I | P60842 | 2,73 | + | 2,49 |  |
| PABPC1 | Polyadenylate-binding protein 1 | P11940 | 2,70 | + | 2,34 |  |
| SPTLC2 | Serine palmitoyltransferase 2 | O15270 | 2,59 | + | 1,24 |  |
| IMPDH2 | Inosine-5-monophosphate dehydrogenase 2 | P12268 | 2,39 | + | 1,76 |  |
| MRPL48 | 39S ribosomal protein L48, mitochondrial | Q96GC5 | 2,25 | + | 0,69 |  |
