# Supplementary material for: Proteome Analysis Reveals Distinct Mitochondrial Functions Linked to Interferon Response Patterns in Activated CD4+ and CD8+ T Cells
Source: Front Pharmacol. 2019 Jul 10;10:727. doi: 10.3389/fphar.2019.00727 (PMC6635586; doi:10.3389/fphar.2019.00727)
Supplement: Table S2 — Significantly upregulated proteins in activated CD8+ T cells. Data show more than two-fold up-regulated (FDR < 0.05) proteins in activated CD8+ T cells, compared to resting CD8+ T cells. Changes marked with a plus (+) are significant (FDR < 0.05). [file Table_2.docx]

**Supplementary Table 2: Significantly upregulated proteins in CD8^+^ T-cells**

| **Gene names** | **Protein names** | **Accession** | **Fold resting CD4+** | **Significant CD4 act vs rest** | **Fold resting CD8+** | **Significant CD8 act vs rest** |
| --- | --- | --- | --- | --- | --- | --- |
| HMGCS1 | Hydroxymethylglutaryl-CoA synthase, cytoplasmic | Q01581 | 9,23 | + | 7,24 | + |
| IRF4 | Interferon regulatory factor 4 | Q15306 | 8,40 | + | 6,40 | + |
| SLC1A5 | Neutral amino acid transporter B(0) | Q15758 | 1,65 |  | 6,10 | + |
| PSAT1 | Phosphoserine aminotransferase | Q9Y617 | 8,09 | + | 5,89 | + |
| TFRC | Transferrin receptor protein 1 | P02786 | 6,32 | + | 5,21 | + |
| SLC7A5 | Large neutral amino acids transporter small subunit 1 | Q01650 | 5,19 | + | 4,88 | + |
| GBP5 | Guanylate-binding protein 5 | Q96PP8 | 6,17 | + | 4,86 | + |
| IL2RA | Interleukin-2 receptor subunit alpha | P01589 | 2,54 |  | 4,58 | + |
| HK2 | Hexokinase-2 | P52789 | 5,81 |  | 4,02 | + |
| TRAF1 | TNF receptor-associated factor 1 | Q13077 | 1,36 |  | 3,73 | + |
| NAMPT | Nicotinamide phosphoribosyltransferase | P43490 | 3,51 | + | 3,35 | + |
